# Heart Failure With Mid-range Ejection Fraction: A Distinctive Subtype or a Transitional Stage?

**DOI:** 10.3389/fcvm.2021.678121

**Published:** 2021-05-25

**Authors:** Qing Zhou, Peixin Li, Hengli Zhao, Xingbo Xu, Shaoping Li, Jing Zhao, Dingli Xu, Qingchun Zeng

**Affiliations:** ^1^State Key Laboratory of Organ Failure Research, Department of Cardiology, Nanfang Hospital, Southern Medical University, Guangzhou, China; ^2^Guangdong Provincial Key Laboratory of Shock and Microcirculation, Southern Medical University, Guangzhou, China; ^3^Bioland Laboratory (Guangzhou Regenerative Medicine and Health Guangdong Laboratory), Guangzhou, China; ^4^Department of Cardiology, Xiangyang Central Hospital, Affiliated Hospital of Hubei University of Arts and Science, Xiangyang, China; ^5^Department of Cardiology and Pneumology, University Medical Center of Göttingen, Georg-August-University, Göttingen, Germany; ^6^State Key Laboratory of Quality Research in Chinese Medicine, Institute of Chinese Medical Sciences, University of Macau, Macau, China

**Keywords:** heart failure, mid-range ejection fraction, preserved ejection fraction, angiotensin receptor-neprilysin inhibitors, sodium-glucose co-transporter 2 inhibitors

## Abstract

Heart failure with mid-range ejection fraction (HFmrEF) was first proposed by Lam and Solomon in 2014, and was listed as a new subtype of heart failure (HF) in 2016 European Society of Cardiology guidelines. Since then, HFmrEF has attracted an increasing amount of attention, and the number of related studies on this topic has grown rapidly. The diagnostic criteria on the basis of left ventricular ejection fraction (LVEF) are straightforward; however, LVEF is not a static parameter, and it changes dynamically during the course of HF. Thus, HFmrEF may not be an independent disease with a uniform pathophysiological process, but rather a collection of patients with different characteristics. HFmrEF is often associated with various cardiovascular and non-cardiovascular diseases. Thus, the pathophysiological mechanisms of HFmrEF are particularly complex, and its clinical phenotypes are diverse. The complexity and heterogeneity of HFmrEF may be one reason for inconsistent results between clinical studies. In fact, whether HFmrEF is a distinctive subtype or a transitional stage between HF with reduced ejection fraction (HFrEF) and HF with preserved ejection fraction (HFpEF) is controversial. In this review, we discuss the clinical characteristics, treatment and prognosis of patients with HFmrEF, as well as the differences among HFmrEF, HFrEF, and HFpEF.

## Introduction

Heart failure (HF) is a serious complication or an end-stage manifestation of various cardiovascular (CV) diseases. It is a complex clinical syndrome with a poor prognosis. Over the last three decades, despite continuous in-depth understanding and considerable progress in HF management, the morbidity and mortality of patients with HF have remained very high, causing a heavy social and economic burden (1, 2).

Historically, the classification of HF is complicated and often confused in different guidelines. Previously descriptive terms of HF include systolic HF, diastolic HF, HF with preserved systolic function, and HF with normal ejection fraction, amongst others (3–7). Since left ventricular ejection fraction (LVEF) is a commonly used parameter to evaluate cardiac function and a significant prognostic predictor of HF, patients with HF are classified into two categories on the basis of LVEF, namely HF with reduced ejection fraction (HFrEF) and HF with preserved ejection fraction (HFpEF) (8, 9). However, the majority of clinical trials on HFrEF or HFpEF exclude patients with a LVEF of between 40 and 50%; this group were once considered as an intermediate group or a “gray-zone” group (8, 9). Interestingly, some characteristics differ between these patients and patients with HFrEF or HFpEF. Therefore, in 2014, Lam and Solomon proposed a new term to describe such patients, namely HF with mid-range ejection fraction (HFmrEF). They pointed out that HFmrEF deserves more attention due to its special clinical, echocardiographic, hemodynamic, and prognostic characteristics (10). Subsequently, HFmrEF was classified formally as a new phenotype of HF in 2016 European Society of Cardiology (ESC) guidelines (11). From then on, clinical studies devoted to HFmrEF have rapidly emerged. However, the results of studies on HFmrEF are not consistent, and are sometimes contradictory, suggesting that HFmrEF may have complex characteristics. Thus, our current understanding of HFmrEF is still insufficient. This leads to a debate about whether HFmrEF is a unique subtype of HF or a transitional stage between HFrEF and HFpEF.

## Definition and Diagnosis

HFmrEF, which previously fell into the category of HFpEF, was once known as “borderline” HFpEF in 2013 American Heart Association/American College of Cardiology Foundation (AHA/ACCF) guidelines (9). HFmrEF was defined as HF with a LVEF of between 40 and 49%, and was listed as a new subtype of HF for the first time in 2016 ESC guidelines (11). According to these guidelines, the diagnosis of HFmrEF includes four elements: HF symptoms with or without signs, LVEF in the range of 40–49%, elevated brain natriuretic peptide (BNP) concentration (>35 pg/ml) or N-terminal pro-B-type natriuretic peptide (NT-proBNP) concentration (>125 pg/ml), and relevant structural heart disease or diastolic dysfunction (11).

Although this definition gives a clear diagnostic cut-off value for LVEF, HFmrEF is not as simple as it seems, because LVEF changes dynamically with an improvement or deterioration in the patient's condition and is not the only parameter used to measure cardiac function (12). Moreover, as the most commonly used technique, echocardiographic measurement of LVEF is not entirely accurate due to possible interobserver and intraobserver variability (13). From this point of view, HFmrEF resembles a container for a crowd of patients with HF with a LVEF of between 40 and 49%. Nevertheless, these patients may have different trajectories and prognoses. Therefore, for further recognition and understanding, HFmrEF can be classified as “HFmrEF improved” or “HFmrEF recovered” (previously a LVEF of <40%), “HFmrEF unchanged” (previously a LVEF of 40–49%), and “HFmrEF deteriorated” (previously a LVEF of ≥50%) based on changes in LVEF over time (14–16). This detailed classification may contribute to a deeper understanding of the pathophysiological process of HFmrEF and partly explain the inconsistent results between clinical studies.

## Epidemiology and Clinical Characteristics

### Prevalence

Based on recent clinical trials and registries, HFmrEF accounts for ~13–24% of HF cases (10, 17–20). For example, in the SwedeHF Registry, which enrolled 42,061 patients with HF, 21% had HFmrEF, whereas 56% had HFrEF and 23% had HFpEF (21). A similar proportion of HFmrEF was observed in the ESC-HF-LT Registry (22). However, the proportion of patients with HFmrEF was inconsistent between studies. In the PINNACLE Registry for first-visit patients with HF, only 7.5% of patients (82,292 of 1,103,386) were classified into HFmrEF category (23).

In addition, data from the GWTG-HF Registry showed that the proportion of patients with HFmrEF was relatively stable over time (between 13 and 15%), whereas the proportion of patients with HFpEF increased from 33 to 39%, and that of patients with HFrEF declined from 52 to 47% (24). In another study examining age-dependent differences in patients with HF, the prevalence of HFmrEF increased slightly with age, whereas the prevalence of HFpEF markedly increased and that of HFrEF significantly decreased (25).

### Demographic Characteristics

Previous cohort and registry studies showed that patients with HFmrEF have intermediate features between those of HFrEF and HFpEF, but closer to those of HFpEF (Table 1) (26–28). However, patients with HFmrEF tend to be younger, and HFmrEF is more common in males compared with HFpEF (18, 19, 21, 22, 26–28).

**Table 1:** Clinical characteristics of patients with HFmrEF compared with patients with HFrEF and HFpEF.

	**GWTG-HF (*****n*** **=** **39,982)**			**SwedeHF (*****n*** **=** **42,061)**			**ESC-HF-LT (*****n*** **=** **9,134)**			**CHART-2 (*****n*** **=** **3,480)**			**ALARM-HF (*****n*** **=** **3,257)**			**OPTIMIZE-HF (*****n*** **=** **37,511)**			**TIME-CHF (*****n*** **=** **622)**		
	**HFrEF**	**HFmrEF**	**HFpEF**	**HFrEF**	**HFmrEF**	**HFpEF**	**HFrEF**	**HFmrEF**	**HFpEF**	**HFrEF**	**HFmrEF**	**HFpEF**	**HFrEF**	**HFmrEF**	**HFpEF**	**HFrEF**	**HFmrEF**	**HFpEF**	**HFrEF**	**HFmrEF**	**HFpEF**
Patients	18,398 (46%)	3,285 (8.2%)	18,299 (45.8%)	23,402 (56%)	9,019 (21%)	9,640 (23%)	5,460 (59.8%)	2,212 (24.2%)	1,462 (16%)	730 (21%)	596 (17.1%)	2,298 (66%)	1,698 (52%)	811 (25%)	748 (23%)	20,118 (53.6%)	7,321 (19.5%)	10,072 (26.9%)	402 (65%)	108 (17%)	112 (18%)
Age, yrs.	79.0	81.0	82.0	72.0	74.0	77.0	64.0	64.2	68.6	66.9	69.0	71.7	–	–	–	70.4	74.3	75.6	75.5	79.0	80.2
Female, %	41.0	51.5	67.6	29.0	39.0	55.0	21.6	31.5	47.9	23.3	28.2	39.2	29.9	35.1	51.6	38.0	52.0	68.0	32.6	46.3	64.3
BMI, kg/m^2^	25.6	26.8	27.3	26.0	27.0	28.0	27.8	28.6	28.4	22.7	22.8	23.2	–	–	–	–	–	–	25.3	25.5	27.0
SBP, mmHg	132.0	141.0	143.0	124.0	131.0	133.0	121.6	126.5	131.0	117.9	124.7	127.9	123.4	139.8	144.9	–	–	–	117.0	127.0	136.0
DBP, mmHg	73.0	74.0	72.0	73.0	74.0	73.0	–	–	–	69.8	71.8	71.9	–	–	–	–	–	–	71.0	73.0	74.0
Heart rate, beats/min	82.0	80.0	79.0	74.0	73.0	74.0	72.9	73.2	72.5	74.0	73.4	71.7	108.5	106.6	108.7	–	–	–	76.0	76.0	74.0
Smoking, %	10.9	8.0	7.4	60.0	55.0	50.0	12.7	10.7	8.1	–	–	–	64.7	58.9	46.1	–	–	–	63.5	60.2	41.1
Hypertension, %	69.9	75.3	77.9	56.0	64.0	72.0	55.6	60.1	67.0	84.7	89.8	91.2	65.5	76.5	71.6	66.0	74.0	77.0	68.9	82.4	85.7
Diabetes mellitus, %	38.3	41.6	38.8	27.0	27.0	28.0	32.3	30.5	29.3	38.1	36.1	33.8	44.0	45.7	41.8	39.0	44.0	41.0	33.6	39.8	39.3
Hyperlipidemia, %	43.5	44.0	40.2	–	–	–	–	–	–	82.2	80.2	78.8	44.7	47.8	39.5	34.0	35.0	31.0	52.2	48.1	36.6
CAD, %	56.8	55.1	43.5	54.0	53.0	42.0	48.6	41.8	23.7	–	–	–	37.8	28.7	20.3	–	–	–	73.9	79.6	63.4
Atrial fibrillation, %	34.5	37.4	38.9	51.0	58.0	63.0	18.3	22.3	32.2	38.1	43.5	51.8	24.2	24.6	26.2	28.0	33.0	32.0	30.0	39.6	42.9
CKD, %	19.4	18.8	17.6	45.0	48.0	56.0	19.5	16.5	19.9	–	–	–	23.1	17.9	18.2	–	–	–	54.0	63.9	61.6
Stroke or TIA, %	14.91	15.98	16.33	–	–	–	9.4	8.3	9.8	18.9	22.1	21.9	–	–	–	–	–	–	14.9	15.7	18.8
Anemia, %	14.73	19.40	20.03	31	35	41	–	–	–	–	–	–	13.2	13.6	14.9	–	–	–	23.6	38.0	34.8
Lung disease, %	25.91	26.87	29.44	28	30	35	15.2	11.6	14.0	–	–	–	22.9	22.4	23.3	–	–	–	20.6	21.3	16.1

*BMI, body mass index; SBP, systolic blood pressure; DBP, diastolic blood pressure; CAD, coronary artery disease; CKD, chronic kidney disease; TIA, transient ischemic attack; HFrEF, heart failure with reduced ejection fraction; HFmrEF, heart failure with mid-range ejection fraction; HFpEF, heart failure with preserved ejection fraction*.

### Etiology

Despite once being considered as a borderline classification similar to HFpEF, HFmrEF shows different etiological features compared with HFpEF. The ESC-HF-LT Registry suggested that the main causes of HFmrEF are similar to those of HFrEF, including ischemic heart disease (IHD) in 41.8% of HFmrEF and 48.6% of HFrEF patients, and idiopathic dilated cardiomyopathy in 27.6% of HFmrEF and 35.1% of HFrEF patients. In contrast, IHD and idiopathic dilated cardiomyopathy account for only 23.7 and 11.6% of patients with HFpEF, respectively (22). Similarly, in the TIME-CHF study, the primary cause of HFmrEF or HFrEF was coronary artery disease (CAD), whereas the primary cause of HFpEF was hypertensive heart disease (18). In the ALARM-HF study, patients with HFmrEF or HFrEF were more likely to be hospitalized for acute coronary syndrome compared with those with HFpEF (20). In addition, previous myocardial infarction was more common in patients with HFmrEF or HFrEF compared with those with HFpEF (29, 30).

In short, IHD is the primary cause of HFmrEF and HFrEF, whereas the underlying diseases of patients with HFpEF often consist of hypertensive heart disease and valvular heart disease. Therefore, from an etiological point of view, patients with HFmrEF are more similar to those with HFrEF rather than HFpEF.

### Comorbidities

In the GWTG-HF Registry, patients with HFmrEF had a similar prevalence of anemia, atrial fibrillation, chronic obstructive pulmonary disease (COPD) or asthma, depression, hypertension, and chronic kidney disease (CKD) compared with those with HFpEF. However, a significantly higher prevalence of IHD was observed in patients with HFmrEF or HFrEF, compared with HFpEF (17). In the ESC-HF-LT Registry, patients with HFmrEF showed a lower incidence of COPD and CKD, compared with the other two groups. An intermediate prevalence of atrial fibrillation in the HFmrEF group was observed. Notably, the incidence of IHD in HFmrEF group was similar to that of HFrEF group, but significantly higher than that of HFpEF group (22). Similar trends in the incidence of IHD among three groups were observed in the MACARF program, TIME-CHF study, and SwedeHF Registry (18, 30, 31). Moreover, patients with HFmrEF or HFrEF carried a higher risk of new IHD events compared with those with HFpEF (30).

In brief, although the characteristics of diseases concomitant with HFmrEF are not consistent in clinical studies, a consistent finding is that patients with HFmrEF have a significantly greater incidence of IHD compared with those with HFpEF (32) (Table 1).

### Prognosis

LVEF is widely considered as an important predictor of CV events in patients with HF. In the CHARM study, when LVEF was <45%, all-cause mortality increased by 39% with every 10% decline in LVEF. With an improvement in LVEF, all-cause mortality and CV death declined. However, once elevated to >45%, an increase in LVEF did not contribute to a further decline in either all-cause mortality or CV death (33). In a meta-analysis, along with an improvement in LVEF, all-cause mortality and CV death declined progressively in patients with HFrEF; however, a similar trend was not observed in patients with a LVEF of ≥40% (34). These findings indicate that LVEF is not an adequate prognostic predictor in patients with HFmrEF or HFpEF.

In a study analyzing the precipitating clinical factors in patients with HF, in-hospital death was significantly lower in patients with HFmrEF compared with those with HFrEF or HFpEF (17). However, in the GWTG-HF Registry, the HFmrEF group showed no difference compared with the other two groups in terms of 5-year mortality. Nevertheless, CV and HF readmission rates were higher in both the HFmrEF group and the HFrEF group compared with the HFpEF group (35).

In the ESC-HF-LT Registry, the 1-year mortality rate of patients with HFrEF, HFmrEF, and HFpEF was 8.8, 7.6, and 6.4%, respectively. By pairwise comparison, there was no significant difference in all-cause mortality of patients with HFmrEF compared with patients with HFrEF or HFpEF. Non-CV mortality in patients with HFmrEF was similar to that of patients with HFpEF, but higher than that of patients with HFrEF. In terms of HF hospitalization rate, the HFmrEF group was similar to the HFpEF group, but significantly lower than HFrEF group (22).

In the SwedeHF Registry, adjusted all-cause mortality in patients with HFmrEF or HFpEF was lower compared with those with HFrEF (21). In the CHART-2 study, patients with HFmrEF showed an intermediate risk of all-cause death, CV death, and hospitalization for HF compared with the other two groups (19).

In terms of patients with acute HF, short-term mortality was lower in patients with HFmrEF or HFpEF, compared with patients with HFrEF in the ALARM-HF study (20). However, in another study of patients suffering from acute decompensatory HF, patients with HFmrEF had similar short-term outcomes compared with those of other categories (36).

In a recent meta-analysis including >600,000 adult patients, patients with HFmrEF demonstrated similar all-cause mortality compared with those with HFpEF, but significantly lower than that of HFrEF patients. Cardiac death was more common in patients with HFpEF, whereas non-cardiac death was significantly more common in the HFrEF group. In addition, no significant differences in all-cause and HF-related hospitalization were observed among the three groups (37).

## Pathophysiology

HF is a complex clinical syndrome with a series of abnormalities in cardiac structure and function. Due to obvious differences in epidemiology, pathophysiology, comorbidity, response to treatment, and prognosis, HFrEF and HFpEF are considered as two distinct pathophysiological entities (38). HFrEF, previously called systolic HF, is generally characterized by impaired left ventricular contractility accompanied by a marked decline in LVEF. The major structural abnormality of HFrEF is eccentric remodeling, followed by progressive ventricular dilatation and volume overload. In contrast, HFpEF, previously called diastolic HF, is predominantly characterized by concentric remodeling accompanied by impaired myocardial relaxation and increased stiffness, resulting in pressure overload (39). In fact, systolic dysfunction and diastolic dysfunction often coexist whether in HFrEF or HFpEF.

Once a component of HFpEF, the exactly pathophysiological mechanisms of HFmrEF remain unclear. According to 2016 ESC guidelines, patients with HFmrEF may have both mild systolic dysfunction and diastolic dysfunction (11). However, this seemingly simple statement may not adequately explain its complex characteristics.

In a recent study of biomarkers in acute HF with different LVEF values, patients with HFmrEF demonstrated an intermediate biomarker feature with interactions between cardiac stretch and inflammation, whereas the biomarker profile of HFrEF was predominantly associated with cardiac stretch and HFpEF with inflammation (38, 40). In another study, epicardial adipose tissue volume was significantly higher in patients with HFmrEF and HFpEF compared to healthy individuals (41). These findings suggested that metabolic and inflammatory mechanisms were involved in the development of HFmrEF.

In the TIME-CHF study, NT-proBNP levels were elevated similarly in the HFrEF group and the HFmrEF group, but were significantly higher than that in the HFpEF group. In addition, NT-proBNP-guided therapy showed similar benefit in HFrEF and HFmrEF, but not in HFpEF, compared with standard therapy (18). In another study, sympathetic activation was greatest associated with adverse outcomes in HFmrEF patients compared with that in HFrEF or HFpEF patients (42). These findings suggested that neurohormonal system activation may play an important role in the pathogenesis of HFmrEF. However, in another study, elevated levels of neuroendocrine hormones including plasma renin activity, aldosterone and norepinephrine were detected in 10% of HFpEF patients, 8% of HFmrEF patients and 21% of HFrEF patients, suggesting neurohormonal activation may only be involved in pathogenesis of a small subset of patients with HFmrEF (43).

In a study evaluating the prognostic value of soluble suppression of tumorigenicity 2 (sST2) in patients with HF, sST2 was an independent predictor of all-cause death and HF rehospitalization for all three groups, indicating that myocardial fibrosis may be a potential pathogenesis of HFmrEF (44). Besides, myocardial dysfunction was also associated with the pathophysiology of HFmrEF (45).

Overall, HFmrEF demonstrates mixed pathophysiological characteristics between HFrEF and HFpEF in existing studies. Although a variety of pathophysiological mechanisms may attribute to the occurrence and development of HFmrEF, extensive data are still lacking, and further studies are required.

## Therapy

Thus far, no prospective studies have specially assessed the effect of pharmacological therapy in patients with HFmrEF. Existing evidences on pharmacological therapy for patients with HFmrEF are based on *post-hoc* analyses of studies that partially or wholly include HF patients with a LVEF of between 40 and 49%, as discussed below.

### Angiotensin-Converting Enzyme Inhibitors/Angiotensin Receptor Blockers

In the OPTIMIZE-HF Registry, ACEI/ARB treatment showed no significant beneficial effects in patients with HF with a LVEF ≥40% (26). In the CHARM-PRESERVED trial, which aimed to assess the effect of candesartan in patients with HF with a LVEF >40%, moderate benefit was observed in preventing HF hospitalization when compared with placebo (46). However, candesartan did not significantly reduce CV death compared with placebo, which may be due to the fact that patients were not classified specially into HFmrEF or HFpEF group (46).

Notably, in a recent analysis using CHARM data to evaluate the effect of candesartan in patients with HF across the entire LVEF spectrum, the HFmrEF group accounted for 17% of all enrolled patients. Candesartan significantly reduced the incidence of CV death or hospitalization in both the HFrEF group and the HFmrEF group, but not in the HFpEF group. Also, candesartan substantially reduced the incidence of recurrent HF hospitalization in patients with HFmrEF (29).

In several studies using data from the SwedeHF Registry, ACEIs/ARBs reduced all-cause mortality in patients with HFmrEF and HFpEF (47–49). Similarly, in a further analysis of the same registry, of 42,061 patients, 21% were classified into the HFmrEF group. ACEIs/ARBs significantly reduced mortality, whether CAD was present or not (21). Similar findings were observed in other studies (18, 19).

In early studies on HFpEF (LVEF ≥40%), ACEIs/ARBs did not demonstrate significant benefit in improving primary outcomes, such as all-cause mortality and CV death. However, subsequent evidence suggested that patients with a LVEF of 40–49% respond differently to treatment compared with those with a LVEF ≥50%. In recent studies specially on patients with HFmrEF, an increasing amount of evidence suggested that ACEIs/ARBs improve clinical outcomes in this group.

In summary, ACEIs/ARBs may be an effective treatment option for patients with HFmrEF. In recent Brazilian Society of Cardiology guidelines, ACEIs or ARBs (if ACEIs are not tolerated) are recommended for patients with HFmrEF (50). Further prospective studies that are focused on this population are required.

### Angiotensin Receptor-Neprilysin Inhibitors

Since the PARADIGM-HF trial was published, ARNIs has been proven to significantly reduce incidence and mortality in patients with HFrEF. Based on this powerful evidence, ARNIs are recommended as a cornerstone pharmacological therapy for HFrEF (11, 51–53). However, the effect of ARNIs in patients with HFmrEF and HFpEF remains unclear.

In the PARAMOUNT trial, ARNIs reduced NT-proBNP levels to a greater extent compared with ARBs. In addition, ARNIs reduced left atrial volume, indicating an improvement in left atrial remodeling (54).

In the subsequent PARAGON-HF trial, which enrolled 4,822 symptomatic HF patients with a LVEF ≥45% and an elevated BNP level, sacubitril/valsartan did not further reduce the risk of total HF hospitalization and CV death compared with valsartan (55). However, in subgroup analyses, a potential benefit was observed in patients with a relatively lower LVEF (45–57%), suggesting that patients with HFmrEF characterized by a mildly reduced LVEF may benefit from sacubitril/valsartan (55, 56). In subsequent analyses based on PARAGON-HF data, pulse pressure and serum uric acid were considered as independent predictors of adverse outcomes in patients with HFpEF, and ARNI reduced pulse pressure and serum uric acid compared with valsartan (57, 58).

In a recent meta-analysis on >5,500 patients, compared with ACEIs and ARBs, ARNIs did not significantly reduce CV death and all-cause mortality. However, ANRIs significantly reduced HF hospitalization and improved physical capacity in patients with HFmrEF or HFpEF. This suggested that ARNIs may reduce HF hospitalization and improve clinical symptoms in patients with HFmrEF or HFpEF (59).

PARALLAX, which is a prospective, randomized, controlled, and double-blind multi-center clinical trial, enrolled patients with HFmrEF and HFpEF to assess the effect of ARNIs on functional capacity (60). In the 2020 ESC Congress-Clinical Trials Hotline Session, the results of the PARALLAX trial were first reported. Compared with individualized medical therapy, ARNIs further reduced NT-proBNP level by 16% at 12 weeks after treatment, and they also significantly reduced the risk of first hospitalization for HF by 51% and of composite events (HF hospitalization, mortality) by 36%.

Given the above evidence, ANRIs may be a useful pharmacological treatment for patients with HFmrEF, as well as patients with HFpEF with a relatively lower LVEF.

### Mineralocorticoid Receptor Antagonists

Mineralocorticoid receptor antagonists have been proven to improve the prognosis of patients with HFrEF. To date, the most important study to assess the effects of spironolactone in patients with HFpEF (LVEF ≥45%) is the TOPCAT study (61). In this study, spironolactone did not significantly improve primary composite outcomes (CV death, aborted cardiac arrest, and HF hospitalization) compared with placebo (61). Interestingly, in a *post-hoc* analysis, a greater potential benefit of spironolactone was observed in patients with a relatively lower LVEF (45–49%) in terms of the primary composite outcome (62), suggesting that patients with HFmrEF may benefit from spironolactone treatment.

Consistent findings were observed in other studies. In a Chinese study examining the role of spironolactone in patients with HFmrEF, spironolactone significantly reduced primary composite outcomes (all-cause death, HF re-hospitalization) compared with placebo (63). In another study, the use of spironolactone at discharge significantly reduced composite outcomes (all-cause death, HF re-hospitalization) in patients with HFmrEF during a mean follow-up period of 2.2 years (64). In a recent meta-analysis of 11 randomized controlled trials (RCTs) with over 4,500 patients, spironolactone treatment reduced HF hospitalization and BNP levels, and improved functional class in patients with HFmrEF or HFpEF (65). These benefits may be partly attributed to alleviation of myocardial fibrosis using spironolactone (65, 66).

Based on these favorable outcomes, mineralocorticoid receptor antagonists are recommended (class IIb) in patients with HFmrEF in recent update to AHA/ACCF guidelines (52, 67).

### Beta-Blockers

Since a large number of RCTs have consistently demonstrated that beta-blockers can significantly improve both short- and long-term outcomes, such as all-cause mortality, CV death, HF hospitalization, and cardiac arrest, these agents are widely recognized as a standard therapy in patients with HFrEF (8, 9, 11, 52). However, whether patients with HFmrEF or HFpEF also benefit from beta-blockers remains unclear.

In the OPTIMIZE-HF Registry, beta-blockers showed no benefit in patients with HFpEF (LVEF ≥40%) (26). Even when the subsequent analysis was refined to patients with a LVEF in the range of 40–49%, beta-blockers did not significantly reduce the risk of mortality and re-admission (68).

Conversely, beta-blockers improved clinical outcomes and reduced mortality in both HFmrEF and HFrEF patients in the CHART-2 study (19). Interestingly, in the SwedeHF Registry, beta-blockers reduced 1-year mortality in patients with HFrEF whether CAD was present or not, but in patients with HFpEF, beta-blockers were only effective in the absence of CAD. In contrast, beta-blockers reduced 1-year mortality in patients with HFmrEF only in the presence of CAD (21). In a meta-analysis of 11 RCTs, beta-blockers were associated with an increased LVEF and improved the prognosis of patients with HFmrEF and HFrEF in sinus rhythm, whereas for patients with atrial fibrillation at baseline, beta-blockers only increased LVEF in the HFmrEF and HFrEF groups, but did not improve prognosis. No significant benefit of beta-blockers was observed in patients with HFpEF whether in sinus rhythm or atrial fibrillation (69). In a nationwide retrospective study, beta-blockers treatment reduced in-hospital mortality in post-acute coronary syndrome patients with HFmrEF (70). However, a recent observational study indicated that beta-blockers did not improve the long-term prognosis in patients with HFmrEF with IHD. Conversely, significant benefits were observed in patients with HFrEF with IHD in terms of long-term outcomes after beta-blockers therapy (71).

In terms of acute HF, in the ALARM-HF study, patients with HFmrEF were intermediate frequently treated with beta-blockers compared with patients with HFrEF or HFpEF (20). In an analysis of data from the KorAHF Registry, beta-blockers improved LVEF in patients with HFmrEF (72).

In brief, according to 2016 ESC guidelines, which recommended that therapy for patients with HFmrEF should be based on the evidence in patients with HFpEF, beta-blockers are not recommended for patients with HFmrEF or HFpEF (11). Similar recommendations were also released in 2017 update to American Heart Association/American College of Cardiology Foundation guidelines (52). However, some studies suggested that beta-blockers may be beneficial for patients with HFmrEF, especially those who have recovered from prior HFrEF after treatment (73, 74). In 2018 Brazilian Society of Cardiology guidelines, beta-blockers are recommended for patients with HFmrEF (50).

### Diuretics

In the SwedeHF Registry, diuretics showed an adverse impact on 1-year all-cause mortality in patients with HFrEF and HFmrEF, but not in patients with HFpEF (21). A similar unfavorable impact on prognosis was observed in the CHART-2 study (19).

Therefore, diuretics are recommended to alleviate symptoms or signs in patients with HFmrEF only in the presence of congestion (11, 52).

### Digoxin

Digoxin is often used as an adjunctive therapy in patients with HFrEF (11). In an analysis of the DIG trial, digoxin reduced HF hospitalization in patients with HFrEF (75). In another study including >11,000 hospitalized patients with HFrEF in the Medicare-linked OPTIMIZE-HF Registry, digoxin reduced HF re-hospitalization, but not all-cause mortality, in older patients with HFmrEF receiving guideline-directed medical therapy (76). Also, in this study, discontinuation of pre-admission digoxin increased the risk of all-cause mortality and the combined endpoint (77).

However, the benefit of digoxin in patients with HFpEF or HFmrEF remains controversial. In a study on 7,374 hospitalized patients with HFpEF in the Medicare-linked OPTIMIZE-HF Registry, the impact of digoxin on short-term (30-day) and long-term (6-year) outcomes was neutral in older hospitalized patients with HFpEF (78). In an observational and multi-center study, digoxin increased the risk of all-cause death and/or re-hospitalization in older patients with HFpEF discharged after acute HF (79).

A retrospective study on the DIG trial included 7,788 patients, 1,195 of whom were diagnosed with HFmrEF. In this group, digoxin reduced primary composite outcomes (CV death or HF hospitalization), mainly reduced HF hospitalization. Interestingly, the effect was greatest in patients with HFrEF, intermediate in patients with HFmrEF, and smallest in patients with HFpEF (80).

### Statins

In early randomized trials, statins did not improve clinical outcomes in patients with HFrEF. In contrast, statins showed a beneficial effect on clinical outcomes, such as mortality, in patients with HFpEF, in the presence or absence of CAD (81–83). The effect of statins in patients with HFmrEF remains unclear.

In the CHART-2 study, statins reduced all-cause mortality in patients with HFpEF, but not in patients with HFrEF or HFmrEF (19). This is consistent with prior studies. However, of note, in the SwedeHF Registry, statin use was associated with a reduction in 1-year mortality in all three groups, irrespective of the presence of CAD (21).

### Sodium-Glucose Co-transporter 2 Inhibitors

Although originally classified as anti-hyperglycemic drugs, SGLT2 inhibitors reduced the risk of HF hospitalization, CV death, and all-cause mortality in patients with HFrEF (84–86). In the 2021 update to the 2017 ACC Expert Consensus Decision Pathway for Optimization of Heart Failure Treatment, addition of SGLT2 inhibitors to standard treatment was recommended to improve clinical outcomes in patients with HFrEF (53). In the newly proposed therapeutic algorithm for patients with HFrEF, simultaneous administration with a beta-blocker and a SGLT2 inhibitor was recommended as the initial treatment (87).

Thus far, whether patients with HFmrEF or HFpEF will benefit from SGLT2 inhibitors remains unclear. Ongoing studies, such as EMPEROR-Preserved, DELIVER, and PRESERVED-HF, will assess the effects of SGLT2 inhibitors in these populations. If the expectations are achieved, SGLT2 inhibitors may be an optional treatment for patients with HFmrEF or HFpEF.

### Other Therapies

Ivabradine is the first selective inhibitor of *I*_*f*_-channel. Due to its benefit in reducing the composite outcomes of mortality or HF hospitalization in patients with HFrEF, it is recommended as an additional therapy to alleviate clinical symptoms and improve outcomes for these patients (11, 52). Heart rate is an essential predictor of clinical outcomes in patients with HF (74). Regarding the importance of heart rate control, ivabradine may also be effective in patients with HFmrEF or HFpEF, but this required further validation.

Tolvaptan is a vasopressin V_2_ receptor antagonist. Its efficacy and safety in patients with HFrEF have been proven in previous studies (88). In a prospective, multi-center, post-marketing surveillance study on 1,741 patients, 286 (16.4%), 795 (45.7%), and 660 (37.9%) patients were categorized as HFmrEF, HFpEF, and HFrEF, respectively. Tolvaptan showed similar benefit in all three groups, suggesting that it may be an effective and safe pharmacological therapy for patients with HFmrEF or HFpEF (88).

Levosimendan is a calcium-sensitizing cardiotonic agent that promotes calcium sensitization of the contractile apparatus without increasing intracellular calcium concentration compared with other inotropes (89). In the LION-HEART multi-center randomized trial, levosimendan reduced plasma NT-proBNP concentration and HF hospitalization, and improved health-related quality of life in outpatients with advanced chronic HF (90). In a recent meta-analysis, intravenous levosimendan was associated with a reduced BNP concentration, an increased LVEF, and reduced short-term mortality in patients with advanced HF (91). Therefore, levosimendan is mainly used in patients with acute HF or chronic decompensated HF. However, no studies have yet investigated the effect of levosimendan in patients with HFmrEF or HFpEF.

Vericiguat is a novel oral soluble guanylate cyclase agonist. It improves myocardial and vascular function by stimulating the activity of guanylate cyclase and increasing the production of cyclic guanosine monophosphate. In the VICTORIA study, which enrolled >5,000 patients with chronic HF and an LVEF of ≤ 45%, vericiguat was associated with a reduced risk of CV death or HF hospitalization (92). However, in the VITALITY-HFpEF randomized trial, 24-week treatment with vericiguat did not demonstrate a beneficial effect on quality of life in patients with HFpEF and recent decompensation (93). Since patients with HFmrEF were partly included in these two studies, whether these patients can benefit from vericiguat remains uncertain; thus, further studies are required in this population.

CDR132L, the first microRNA-132 inhibitor, is a synthetic special antisense oligonucleotide. In preclinical models, CDR132L demonstrated beneficial effects on improving and even reversing HF. In the first-in-human study of CDR132L, which enrolled patients with a LVEF in the range of 30–50% or an NT-proBNP concentration of >125 ng/L, CDR132L improved cardiac function and ameliorated cardiac fibrosis (94). CDR132L may be a promising drug for patients with HFmrEF or HFrEF; however, this requires further validation.

Iron deficiency is prevalent in patients with HFrEF, HFpEF, and HFmrEF. Progression of iron deficiency accelerates HF deterioration (95). Intravenous iron treatment improved exercise capacity, relieved HF symptoms, and improved quality of life in patients with HFrEF and iron deficiency (96). However, whether patients with HFmrEF or HFpEF patients can benefit from intravenous iron remains uncertain (97).

In general, despite HFmrEF have intermediate features between HFrEF and HFpEF, patients with HFmrEF demonstrate a comparable response to guideline-directed medical therapies as patients with HFrEF (Table 2).

**Table 2:** Treatment response of patients with HFrEF, HFmrEF, and HFpEF.

	**ACEI**	**ARB**	**ARNI**	**MRA**	**Beta-blocker**	**SGLT2 inhibitor**	**Statins**
HFrEF	++	++	++	++	++	++	?
HFmrEF	+	+	++	+	+	?	?
HFpEF	/	+	++	+	/	?	++

*ACEI, angiotensin-converting enzyme inhibitor; ARB, angiotensin receptor blocker; ARNI, angiotensin receptor-neprilysin inhibitor; MRA, mineralocorticoid receptor antagonist; SGLT2 inhibitor, sodium-glucose co-transporter 2 inhibitor; HFrEF, heart failure with reduced ejection fraction; HFmrEF, heart failure with mid-range ejection fraction; HFpEF, heart failure with preserved ejection fraction. The symbol +, ++, /, ? represent moderately effective, significantly effective, noneffective, and probably effective, respectively*.

## Similarities and Differences Between Acute HFmrEF and Chronic HFmrEF

Since the majority of studies on HFmrEF enrolled patients with chronic HF (CHF), studies specially for HFmrEF patients with acute HF (AHF) were relatively few.

In current studies on HFmrEF patients with AHF, the proportions of HFmrEF patients were ~14–25% (20, 36, 98). These patients demonstrated intermediate features between HFrEF patients and HFpEF patients. HFmrEF patients were older and more commonly male compared with HFrEF patients, whereas they were younger and more likely to be female compared with HFpEF patients. Similar characteristics were observed in patients with CHF (18, 20, 22).

In terms of biomarkers of AHF patients, the HFmrEF group also showed intermediate characteristics between the other two groups (40). However, in CHF patients, HFmrEF resembled more closely HFrEF except lower BNP level (99). In addition, some biomarkers played an important role in prognostic prediction. For example, elevated BNP level predicted an increased risk of mortality in all three groups (100). The difference was that in AHF patients, the prognostic significance of BNP was higher in HFrEF compared with that in HFmrEF and HFpEF (100), while in CHF patients, BNP was most closely associated with the prognosis of the HFmrEF group compared with other two groups (99).

Considering the etiological aspect, IHD was the leading cause of HFmrEF patients whether with AHF or CHF. From this viewpoint, HFmrEF was closer to HFrEF but not HFpEF. However, regarding short-term mortality, HFmrEF patients showed a lower risk compared with HFrEF patients, but a similar risk to HFpEF patients (20, 98). However, in discharge AHF patients, the long-term all-cause mortality of all three groups was comparable high (98).

Regarding pharmacological treatment, neurohormonal activation was associated with an increased risk of all-cause mortality and CV death in HF patients. Previous studies showed that this association was greatest in HFmrEF patients, while it was weakest in HFpEF (42). These findings suggested that neurohormonal therapies may be effective for HFmrEF patients, which was consistent with observations in clinical trials, such as SwedeHF registry (21). However, in acute HFmrEF patients receiving guideline-directed medical therapy, only beta-blockers showed favorable effect on in-hospital mortality, whereas ACEIs/ARBs and MRAs did not improve outcomes (98). Therefore, further studies are required to evaluate the effect of ACEIs/ARBs or MRAs in acute HFmrEF patients.

## Transitions Among the Three HF Groups

According to LVEF, recent clinical guidelines classify HF into three groups: HFrEF, HFpEF, and HFmrEF (11). As a gray zone between HFrEF and HFpEF, this new definition has encouraged research into the potential characteristics, pathophysiology, and treatment of HFmrEF (101). Of note, despite LVEF is widely used as the basis for classifying HF in recent guidelines (9, 11), it is not a precise indicator of cardiac function, which may be influenced by many factors. For example, LVEF may provide imprecise implications in the presence of mitral regurgitation, aortic stenosis, or ventricular hypertrophy (102). In addition, there is substantial variability among different imaging techniques for LVEF measurement (103). Even when using the same imaging method, interobserver variability may exist. Especially noteworthy is the fact that LVEF is a dynamic index and may increase or decrease during the course of HF. In several studies, transitions in LVEF were observed (12, 104–106), suggesting that the cut-off value of LVEF is artificial, and LVEF may change dynamically over time. In other words, transitions among these three groups require more attention rather than a static LVEF value.

**Figure 1:**
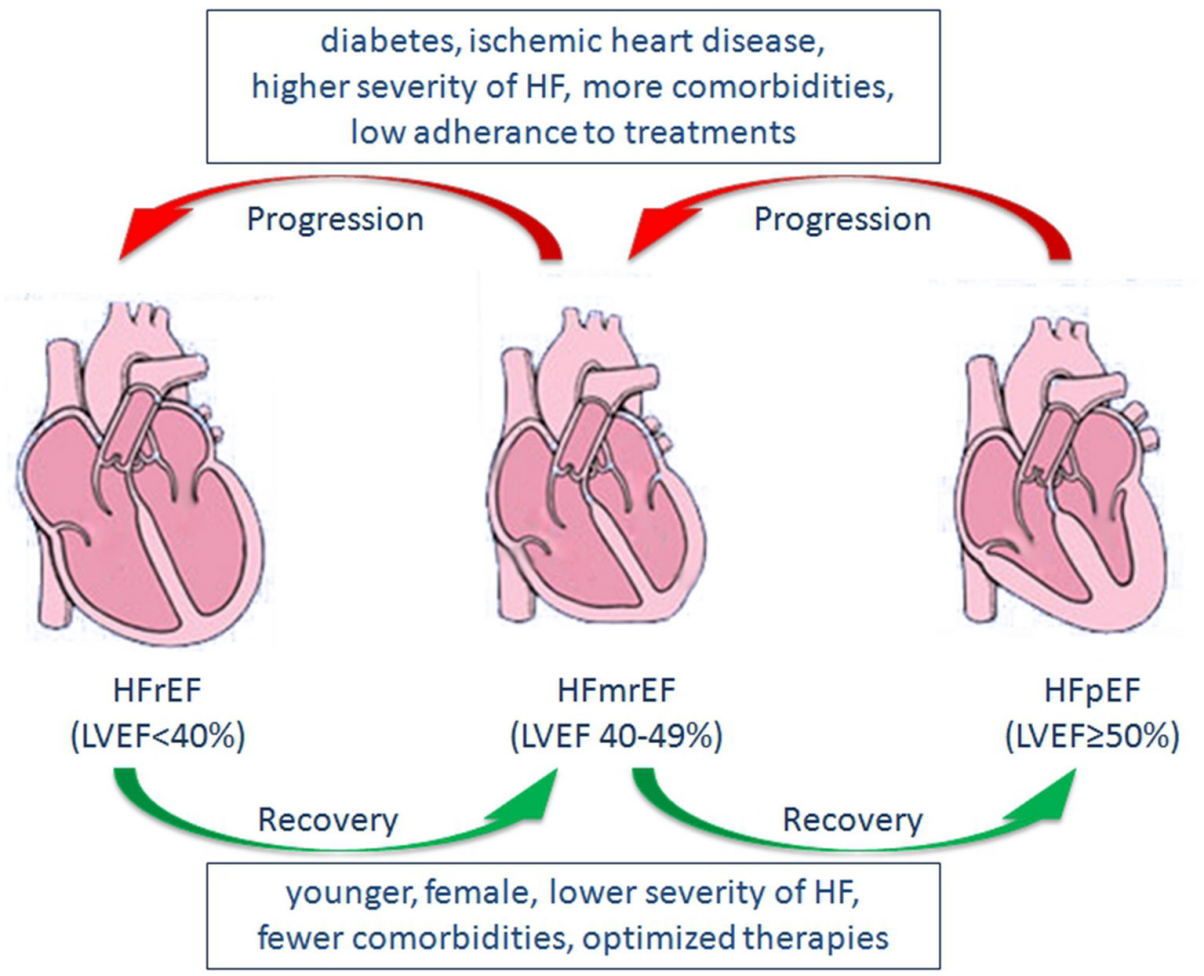
Predictors of changes in LVEF, and Transitions among HFrEF, HFmrEF, and HFpEF. LVEF, left ventricular ejection fraction; HFrEF, heart failure with reduced ejection fraction; HFmrEF, heart failure with mid-range ejection fraction; HFpEF, heart failure with preserved ejection fraction; HF, heart failure.

In a cohort study examining the natural history of LVEF over time in patients with HF, patients who suffered from previous myocardial infarction were more likely to transition from HFpEF to HFrEF, whereas females and those using beta-blockers tended to transition from HFrEF to HFpEF (105). Similarly, in a community-based cohort study, average LVEF decreased by 5.8% over 5 years in patients with HFpEF, and a greater decline was observed in older individuals and individuals with CAD. In contrast, average LVEF increased by 6.9% over 5 years in patients with HFrEF, and a greater increase was observed in females, younger patients, individuals without CAD, and those receiving guideline-directed medical therapy (12). In a recent study evaluating the prognostic implications of longitudinal LVEF change in HF, transitions among the three groups were observed during follow-up. Increases in LVEF occurred in 25% of HFmrEF patients and 26% of HFrEF patients, whereas decreases in LVEF occurred in 39% of HFpEF patients and 37% of HFmrEF patients (107). Predictors of increased LVEF included younger, female, lower severity of HF, fewer comorbidities, optimized therapies, and predictors of decreased LVEF included diabetes, IHD, higher severity of HF (107, 108) (Figure 1). Moreover, a decrease in LVEF over time is associated with increased mortality and/or HF hospitalization, whereas an increase in LVEF is associated with reduced mortality and/or hospitalization (12, 107, 108).

Considering the trajectory of LVEF over time, HFmrEF may occur either as a recovery from HFrEF, or a deterioration from HFpEF. Also, it may be the initial presentation of patients with HF (109). Thus, HFmrEF represents a large group of patients with heterogenous features and consists of at least three subgroups, including HFmrEF improved, HFmrEF unchanged, and HFmrEF deteriorated (15, 16). Although both are categorized as HFmrEF, HFmrEF improved (an increase in LVEF after treatment for prior HFrEF) may have a distinct pathophysiological process, treatment response, and prognosis compared with HFmrEF deteriorated (declined LVEF from prior HFpEF) (Figure 1). In a recent study examining the epidemiology, pathophysiology and clinical outcomes of HFmrEF, HFmrEF improved patients showed significantly better clinical outcomes compared with HFmrEF deteriorated individuals, whereas no significant differences were observed in clinical outcomes between the HFmrEF deteriorated group and matched patients with HFpEF (110). Similar findings were observed in the CHART-2 study (19).

In summary, despite a universal diagnosis of HFmrEF, patients may have different characteristics, pathophysiological features, clinical courses and prognoses according to diverse changes in LVEF (107, 111). By recognizing the continuous spectrum of HF and the limitations of LVEF, we should pay attention to the trajectory of LVEF over time, refine the classification of HF based on pathophysiological homogeneity rather than LVEF value alone (112–114), and design an individualized, evidence-based therapeutic strategy (50, 114).

## Conclusion

As a new HF classification, HFmrEF demonstrates intermediate characteristics between those of HFrEF and HFpEF. Whether HFmrEF represents a distinct subtype of HF or is a transitional stage between HFrEF and HFpEF remains controversial. In terms of the longitudinal trajectory of LVEF and transitions among the three HF groups, HFmrEF resembles a transitional stage between HFrEF and HFpEF rather than a unique subtype, including patients who have recovered from previous HFrEF, patients who have deteriorated from previous HFpEF, and patients with a relatively stable LVEF in the range of 40–50%. More importantly, different LVEF trajectories of patients with HFmrEF often indicate different prognoses. A refined classification may be helpful to further understand the clinical characteristics and pathophysiology of HFmrEF, and to make optimized and individualized treatment decisions.

## Author Contributions

All authors listed have made a substantial, direct and intellectual contribution to the work, and approved it for publication.

## Conflict of Interest

The authors declare that the research was conducted in the absence of any commercial or financial relationships that could be construed as a potential conflict of interest.
